# Seizure control in patients with epilepsy: the physician vs. medication factors

**DOI:** 10.1186/1472-6963-8-264

**Published:** 2008-12-18

**Authors:** Jerzy P Szaflarski, Angela Y Rackley, Christopher J Lindsell, Magdalena Szaflarski, Stephen L Yates

**Affiliations:** 1 Department of Neurology and the Cincinnati Epilepsy Center, University of Cincinnati Academic Health Center, Cincinnati, OH, USA; 2 Department of Emergency Medicine, University of Cincinnati Academic Health Center, Cincinnati, OH, USA; 3 Department of Public Health Sciences, Family Medicine, and the Institute for the Study of Health, University of Cincinnati Academic Health Center, Cincinnati, OH, USA; 4 UCB, Inc., Medical Affairs, Smyrna, GA, USA

## Abstract

**Background:**

Little is known about the relationship between types of healthcare providers and outcomes in patients with epilepsy. This study compares the relative effects of provider type (epileptologist vs. other neurologist) and pharmacologic treatment (newer vs. older antiepileptic drugs) on seizure control in patients with epilepsy.

**Methods:**

We conducted a retrospective study of patients with medication-resistant epilepsy. Consecutive charts of 200 patients were abstracted using a standard case report form. For each patient, data included seizure frequency and medication use prior to, and while being treated by an epileptologist. Changes in seizure frequency were modeled using a generalized linear model.

**Results:**

After transferring care from a general neurologist to specialized epilepsy center, patients experienced fewer seizures (p < 0.001) and were more frequently seizure-free (p < 0.001). The improved seizure control was not related to treatment with newer vs. older antiepileptic drugs (p = 0.305).

**Conclusion:**

Our findings suggest an association between subspecialty epilepsy care and improved seizure control in patients with medication-resistant epilepsy. Further research should prospectively determine whether patients with medication-resistant epilepsy would benefit from being routinely referred to an epilepsy specialist.

## Background

In recent discussions about health care cost and utilization in the United States, several factors emerge as reasons for cost escalation.[1,2] Among them are increased use of specialty physicians instead of generalists, choice of expensive procedures instead of investment in preventive measures, high-cost of new technologies and pharmaceuticals, aging of the population and increasing health care and administrative costs.[3] The *perceived *need for more specialists to provide increasingly complex care, especially in the extremes of life has led to proliferation of specialists over the last 40 years as demonstrated by the 22% reduction in the number of primary care physicians (PCP; from 50% to 28%) despite the concurrent increase of the total number of physicians (from 250,000 to 650,000).[4] However, evidence shows that the number of specialists does not always lead to improved care. Based on studies of cancer, heart disease, stroke, and self-related health appropriate supply of PCPs is thought to be associated with improved health outcomes.[5] One study has documented a reverse relationship between the overall quality of care received by Medicare beneficiaries and the cost of care, with this negative relationship being driven by the use of intensive and costly care that crowded out the use of the more effective preventive and general medical care.[6] Another study found that although cardiologists improved the outcome of patients with myocardial infarction when compared to the care provided by non-cardiologists, the best outcome was achieved when cardiologists and generalists combined their efforts.[7] A similar relationship has been shown in neurologic vs. general medical care of patients with acute stroke; neurologic care was more costly but led to substantially improved outcomes.[8] One possible explanation for this disparity between cost and quality of care is that while costly care and procedures are overutilized, less expensive measures, such as prevention, are underutilized.[6,8]

Studies examining the relationship between quality of care and specialized care in patients with epilepsy are limited. One recent investigation explored the impact of an epilepsy clinic on seizure control in institutionalized patients and found that treatment by an epileptologist led to an overall favorable outcome; 55% of patients experienced reduction in seizure frequency and 23% of them became seizure-free.[9] Another small retrospective study also found improved seizure control in patients referred to an epilepsy center.[10] Considering multiple, potential factors in the relationship between quality and cost of care in patients with epilepsy, we conducted a retrospective chart review study to determine what factors contribute to seizure control in patients with epilepsy across various clinical settings. Specifically, we aimed to compare the relative contribution of type of provider (epileptologist vs. general neurologist) and pharmacologic treatment (newer vs. older antiepileptic drugs, or AEDs) to the overall seizure control in patients with medication-resistant epilepsy. We hypothesized, based on previous research, that patients managed by non-epileptologists would have poorer seizure control than when they were subsequently managed by epileptologists, and that this difference would depend on the type of physician and not on the choice of AED. [9-11]

## Methods

### Subjects

This retrospective observational study was approved by the Institutional Review Board of the University of Cincinnati (consent requirement waived). All patients treated in the outpatient clinic of the Cincinnati Epilepsy Center between 11/1/06 and 8/31/07 were eligible to participate in the study. Overall, we enrolled 200 patients, with balanced numbers of patients from different clinical settings to eliminate the possibility of the clinical setting confounding the results – 100 institutionalized patients (e.g., nursing home, group home, or independent supervised living) and 100 non-institutionalized patients.

### Data collection

All potential study subjects were identified by a study coordinator who was blinded to the overall goals of the study. We first identified institutionalized patients and then non-institutionalized patients matched for age and gender from the group of patients evaluated by the same physician on the same day. Next, the selected charts were reviewed and abstracted by a study physician. Charts were abstracted by a single investigator (AYR) using a standardized case report form and data dictionary with explicit, pre-specified data definitions. Disease- and treatment-specific data including type of epilepsy, AED therapy before and during the treatment in the epilepsy center, duration of epilepsy, outcomes (as defined below), and complications of treatment were extracted. In cases where the chart entry was not clear, the physician responsible for the patient was directly contacted with questions and/or a request for additional records (approximately 5% of the charts). Clinical notes were also reviewed for the clinician's impression regarding response to therapy and to ascertain side effects of AED treatment. Approximately 25% of charts were selected at random and re-reviewed by another investigator (JPS) to confirm the accuracy of the collected data.

### Data definitions

For the purpose of this study, we defined epileptologist as any neurologist who had undergone specific training in the diagnosis and management of epilepsy (i.e., completed at least one year of epilepsy/clinical neurophysiology fellowship) and whose scope of practice was focused on the management of patients with epilepsy at the time of the study. All other neurologists were considered non-epilepsy (or other) physicians.

We defined the following seizure outcome variables:

1) ***Baseline seizure frequency***: Seizure frequency at the time of the initial visit in the epilepsy center was defined as the average number of seizures per month at the time of the first visit spanning the 3 months prior to the visit; expanding the "*baseline seizure frequency*" variable to 12 months to match the "current mean monthly seizure frequency" variable was impractical as the majority of charts did not contain such information.

2) ***Current mean monthly seizure frequency***: Defined as number of seizures per month averaged over 12 months prior to the most current visit. This variable was treated either as categorical (presence or absence of seizures, also called the "*seizure-freedom" *variable) or continuous (average number of seizures per month).

Duration of epilepsy was defined as the difference between the age at epilepsy onset and the age at the last visit; two additional epilepsy duration treatment variables were defined: the duration of treatment by an epileptologist, measured as the number of years the patient was followed in the Cincinnati Epilepsy Center, and the duration of treatment by a non-epileptologist, measured as the number of years the patient was treated by a general neurologist(s) for epilepsy before referral to the epilepsy center. Some patients either self-referred or were referred by their primary care specialist, but all patients included in this study were followed by a general neurologist prior to their first visit to the epilepsy center.

Medication outcome variables were defined as the number of "older AEDs" (bromides, barbiturates, benzodiazepines, phenytoin/other hydantoins, carbamazepine immediate release, valproic acid, succinamides) and "newer AEDs" (carbamazepine extended release formulations, felbamate, gabapentin, lamotrigine, levetiracetam, oxcarbazepine, pregabalin, tiagabine, topiramate, zonisamide, investigational drugs) ever used. Carbamazepine extended release was included in the "newer AEDs" category as this formulation was introduced to the market along with the other "newer AEDs"; this AED has been shown to be more efficacious and causing less side effects than the immediate release carbamazepine.[12] Other medication variables included the number and type of AEDs, and whether they were prescribed by an epileptologist or other neurologist. In cases when it was not clear who prescribed the AED or when the AED was initiated by one physician but adjusted by the epileptologist, both were credited with the use of the particular AED. Finally, we broadly defined AED side effects as the presence or absence of side effects leading to AED discontinuation.

### Data analyses

Data were initially characterized using descriptive statistics (means and standard deviations or frequencies and percentages as appropriate). We compared between baseline and the most current visits using Fisher's Exact test for categorical data, and the Wilcoxon Signed-Rank test for continuous data. We modeled the effect of provider type and medication use on change in seizure frequency using a generalized linear model. No adjustment for covariates or multiple comparisons was attempted due to the exploratory nature of the study (see below). All data management and analyses were performed using SPSS V. 15.0 (SPSS Inc., Chicago, IL, USA), the significance level was set to 5% (*α *= 0.05) for all analyses.

## Results

Twenty-two subjects were excluded from analyses because they were followed by an epileptologist for less than 1 year, and the current mean monthly seizure frequency could therefore not be assessed. Demographic and clinical characteristics of the included subjects are presented in Table 1. Overall, the median age for the studied population was 43 years; 45.5% of patients were female. Approximately 14% of patients were black. The median age of epilepsy onset was 10 years and the median duration was 30.5 years. The epilepsy diagnosis distribution was similar to major epidemiological studies and included 34.8% patients with generalized epilepsies (idiopathic and symptomatic combined) and 57.3% of patients with focal onset epilepsies.[13] In approximately 7.9% of patients it was not clear whether they had focal or generalized epilepsy onset. The treatment by epileptologists was shorter than the treatment by other physicians (6 years vs. 22 years). Despite that, more patients achieved seizure freedom while treated by epileptologists (26.4% vs. 10.1%; Table 1).

**Table 1:** Demographic and clinical characteristics of patients included in the study.

Age in years (median, range)	43 (21–83)
Female (%)	54.5

Black (%)	14.0

Age at the seizure onset in years (median, range)	10 (0–81)
Epilepsy duration in years (median, range)	30.5 (2–66)

Epilepsy type	
Generalized, including symptomatic generalized epilepsy (%)	34.8
Focal onset (%)	57.3
Unknown (%)	7.9

Duration of treatment by an epilepsy specialist in years (median, range)	6 (1–29)
Duration of treatment by physician(s) other than epilepsy specialist in years (median, range)	22 (1–62)

Average number of seizures/month prior to treatment by an epilepsy specialist (median, range)	4 (0–3000)
Average number of seizures/month over the last 12 months (median, range; the "*seizure freedom*" variable)	1 (0–300)

Seizure-free at the time of the initial visit (%)	10.1
Seizure-free at the time of the last visit (%)	26.4

Psychiatric comorbidities (%)	33.7
Neurologic comorbidities (%)	75.8
Stroke	7.9
Tumor	6.7
Trauma	6.2
Cerebral palsy/mental retardation/developmental delay	46.6
Other neurologic comorbidities	30.3

MRI ever done (%)	74.7
DEXA done (%)	28.7

Receiving bone replacement therapy (%)	39.9
Patients with falls (%)	7.3
Patients with fractures (%)	6.7

Epileptologists prescribed fewer AEDs than other physicians (median of 2 versus a median of 5, p < 0.001) – this is likely related to a much shorter period the patients were treated by epileptologists vs. other physicians (6 vs. 22 years). We noted differences in medication side effects leading to medication discontinuation (newer AEDs were discontinued less frequently than old AEDs; p = 0.043). Further, we noted minor differences in types of AED used between the epileptologists and other physicians. Epileptologists used pregabalin (8.4% v 2.2%; p = 0.002) more frequently, while the other physicians used bromides (4.5% v 0.6%; p = 0.045), carbamazepine (55.1 v 10.7; p = 0.001), carbamazepine extended release (17.4 v 11.8; p = 0.001), chronic benzodiazepines (25.8% v 12.4%; p < 0.001), felbamate (16.3 v 7.9; p = 0.013) and zonisamide (10.1 v 8.4; p < 0.001) more frequently. It is noted that there were fifteen comparisons for this analysis, so accepting the significance level of 0.05 might lead to an increase in Type I error (false positive finding). Using the Bonferroni correction, a conservative significance level that preserves the Type I error would be 0.0033.

We noted that the proportion of seizure-free patients increased significantly over the time the patients received treatment in the epilepsy center, from 10.1% to 26.4% (p < 0.001). During the same time, the median seizure frequency decreased from 4 seizures per month to 1 seizure per month (p < 0.001).

We recognize the particular problems that can arise when analyzing skewed data using a parametric statistical model. To enable the use of parametric models, we computed the change in seizure frequency as the primary outcome variable, rather than an absolute seizure number, since this was more likely to be normally distributed. Second, we removed outliers to avoid significantly impacting the model by including extreme values. To validate our assumptions, we checked the distribution of residuals from the general linear model and found them to be close to normally distributed, suggesting transformation of the data was not strictly necessary. Thus, using a generalized linear model to determine whether the epileptologist(s) prescribing newer vs. older AEDs might account for the change observed when care was transitioned from the generalist, we found that whether older or newer, or a combination of older and newer drugs, was given, it did not significantly change the seizure frequency (p = 0.305).

## Discussion

In this retrospective study we evaluated the contribution of medication choice and physician specialty on seizure control in a group of patients with medication-resistant epilepsy. We found that patients treated by epileptologists fared better than when they were previously treated by other neurologists, and that the improved seizure control was not related to whether newer AEDs were prescribed in preference to older AEDs. In other words, we postulate that patients owe improved seizure control to epileptologists' better ability to refine treatment vis-à-vis other neurologists.

One focus of the current health care debate is whether we should invest in specialty care or general care.[1,2] In the neurology setting, several examples of improved outcomes are noted when neurologists manage patients with acute or chronic conditions when compared to generalists (same relationship is noted between neurology sub-specialists and generalists). For example, implementation of a general neurology care line has led to decreased hospital admissions and improved quality of the provided services when compared to other non-specialty care for neurological patients.[14] In neurology, subspecialty care may lead to further improvement. Overall, the most frequently noted positive influence of subspecialty care on outcomes is for acute stroke patients. In one study, neurologists treating patients with acute stroke attained better outcomes when compared to internists or family practitioners, even after adjusting for patient age, co-morbidity, and other characteristics. The increased costs associated with providing subspecialty care were justified, in the authors' opinion, since subspecialty-treated patients had lower mortality and higher chance of discharge to rehabilitation facilities possibly decreasing the long-term cost (not measured in the study). Better outcomes were thought to be possibly related to specialized training, improved ability to identify stroke mechanisms, and provision of more targeted care.[8] Multiple studies have confirmed these findings, and the fact that stroke units manned by stroke specialists achieve better treatment outcomes when compared to patients not treated in stroke units is not a surprise.[15] Similarly, the recently created Parkinson's Disease Research Education and Clinical Centers (PADRECC) expect better outcomes in patients with Parkinson's disease who are treated in centers specializing in the management of patients with movement disorders as similar programs in geriatrics and mental illness have already led to improved outcomes for patients cared for by geriatricians and psychiatrists, respectively.[16]

The data on the effectiveness of neurologists overall and epileptologists in particular in improving non-surgical outcomes of patients with epilepsy is limited. Further, a recent study showed that about 35% of non-institutionalized patients with the diagnosis of epilepsy are not actively treated by neurologists or epilepsy specialists.[17] This underscores the importance of examining whether the care provided by epilepsy specialists over other physicians provides epilepsy patients with incremental benefit. Further, one large study conducted in the United Kingdom surveyed patients' perceptions of the care available for epilepsy. Although satisfaction was overall high, there was a perceived lack of interface between general and epilepsy care; establishment of epilepsy care centers, in order to provide improved care to the epilepsy patients, was suggested.[18] Finally, an extensive review of nursing literature did not find any evidence that specialist epilepsy nurses improved outcomes for epilepsy patients, and a recent Cochrane review did not find any peer reviewed studies evaluating whether there was any relationship between the quality of care provided by epileptologists vs. general neurologists or general practitioners. [19,20]

In terms of quality of care in epilepsy, one study found that epileptologists better recognize epilepsy syndrome(s) than other physicians, and are able to simplify medication schedules while their patients overall achieve better seizure control.[9] Our study confirms that long-term care by an epileptologist improves seizure control in many patients, including patients with multiple handicaps. Another study evaluated the patterns of patient referral to the epilepsy centers and found that only 27% were referred to the epilepsy centers by general neurologists, while all other patients were referred by non-neurologists; 41% of the patients wished they were referred earlier. Unfortunately, these authors did not assess the outcomes of treatment after the transition of care.[11] Finally, we recently conducted a small study on a convenience sample of epilepsy patients who transitioned their care from general neurology practices to an epilepsy center due to insurance changes. We observed significant improvements in seizure-freedom (48.5% vs. 69.1%; p = 0.03) between the time of the first and last visit, suggestive of improved outcomes after receiving care in an epilepsy center.[10] Although improvements in outcomes were noted, the reasons for the improvements were not investigated. Therefore, it appears that the current study is the first to focus on the benefits of being treated for epilepsy by an epileptologist when compared to a non-epileptologist, accounting for the possible treatment differences in various environments. We show that epileptologists provide significant benefits to patients with medication-resistant epilepsy beyond what can be offered by non-epileptologists. This suggests that patients with medication-resistant epilepsy may benefit from earlier referral to an epilepsy center for further diagnosis and management as improved seizure control is associated with better quality of life and better ability to integrate with the society. [21-24]

Several limitations of the study should be noted. The retrospective design of the study introduces potential biases, including patient selection and possibly incorrect ascertainment of clinical data such as seizure frequency. It is also possible that the initial seizure frequency may be exaggerated since a sudden or transient escalation in seizure frequency may have been a reason for patient referral to an epilepsy specialist. We attempted to minimize this by averaging the initial seizure frequency over three months, a time 3–6 times longer than our current waiting time for patients to see an epilepsy specialist. Further, ascertainment of seizures in non-institutionalized patients may be subjected to another patient-related bias – these patients may not report their seizures as such reporting may lead to driving and other restrictions with significant effects on employment and quality of life.[22,25] Lack of data that would potentially explain the reasons for improved seizure control in patients treated by epileptologists limits interpretation; we did not collect data on re-classification of seizure diagnosis or whether syndrome-specific AEDs were used or not by general neurologists (e.g., valproic acid in patients with IGE). Further prospective studies addressing these issues will be needed.

In summary, our study shows that patients with medication-resistant epilepsy may benefit from evaluation and treatment by an epileptologist, with the primary benefit being reduced seizure frequency. This retrospective study should stimulate prospective evaluations focusing on the quality and cost of care in patients with epilepsy and investigations of whether the higher costs of specialty epilepsy care are offset and justified by improvements in quality of life.

## Competing interests

In the last 5 years, Dr. Jerzy P. Szaflarski received grants, honoraria for speaking and consulting fees from UCB, Inc.; Dr. Angela Y. Rackley received a grant from UCB, Inc.; Dr. Magdalena Szaflarski declares that she has no competing interests; Dr. Christopher Lindsell declares that he has no competing interests; Dr. Stephen Yates in an employee of UCB, Inc. This study was supported but not commissioned by UCB, Inc. Young Investigator Mentored Research Project Award to AYR and it was presented in part at the Annual Meeting of the American Epilepsy Society, November 30 – December 4, 2007, Philadelphia, PA

## Authors' contributions

JPS and AYR have conceived the study and obtained funding, participated in its design and coordination, and drafted the manuscript. CJL, MS and SLY have participated in study design and data analysis; they reviewed the manuscript.

## Pre-publication history

The pre-publication history for this paper can be accessed here:

http://www.biomedcentral.com/1472-6963/8/264/prepub
